# Nondestructive Testing in Food Quality and Safety: Development and Applications

**DOI:** 10.3390/foods14132339

**Published:** 2025-07-01

**Authors:** Mingfei Pan

**Affiliations:** State Key Laboratory of Food Nutrition and Safety, Tianjin University of Science and Technology, Tianjin 300457, China; panmf2012@tust.edu.cn

## 1. Introduction

Food quality and safety have consistently been a central global concern, directly related to public health and well-being, as well as to the sound development of the food industry [1,2,3]. As consumer demands for food quality grow increasingly stringent, the limitations of traditional inspection techniques in dealing with complex and changing food matrices, as well as increasingly hidden quality problems, are becoming more and more prominent, making it challenging to achieve the high standards required for accuracy, sensitivity, and efficiency. The emergence of nondestructive testing (NDT) technologies has revolutionized the food quality and safety inspection landscape. These advanced techniques can assess food products without causing any damage, ensuring the integrity of food and its safety for consumption [4,5].

NDT techniques include a wide range of methods such as visual inspection, sensory evaluation, physical measurements, chemical analysis, and microbiological testing [6,7]. These methods are specifically designed to detect defects, contaminants, and other quality issues in food products (such as spoilage, foreign objects, and pathogenic microorganisms). Technologies such as spectroscopy (near-infrared spectroscopy, Raman spectroscopy, and terahertz spectroscopy), ultrasonic testing, nuclear magnetic resonance, X-ray-computed tomography, laser scattering, electronic nose, and optical methods have been developed to analyze food products for various purposes [8,9,10]. Novel biosensing, machine vision, and image processing also play a key role in food quality and safety inspection for detecting surface defects, foreign objects, and other contaminants [11,12,13]. These nondestructive technologies are essential for ensuring public health and maintaining consumer confidence in the food industry.

## 2. An Overview of the Published Articles

The research by Pang et al. (Contribution 1) innovatively proposed a nondestructive detection method termed “Dice Loss Improved Self-Supervised Learning-Based Prototypical Network (Proto-DS)”. This method addressed the challenge of the effective classification of small-scale imbalanced datasets, while minimizing deviations in dominant classes. By reducing reliance on common categories, it not only mitigated label bias but also enhanced the model confidence. Notably, this model demonstrated significant advantages in the spectral data collection of food products such as Citri Reticulatae Pericarpium (Chenpi), Chinese herbal medicines, and coffee beans. Remarkably, the Proto-DS model achieved an average accuracy rate of nearly 90% across varying sample quantities, surpassing traditional logical models. Consequently, the self-supervised learning approach introduced in this study can improve the performance of imbalanced learning. Furthermore, combining prototype networks with dice loss offers a potential solution for constructing efficient models with limited training data.

In the work of Zhan et al. (Contribution 2), a fluorescence hyperspectral imaging system (FHIS) was employed to integrate with machine learning algorithms to predict the sucrose concentration in apples, thereby enabling the assessment of apple quality. The two prominent fluorescence characteristics exhibited by apples under fluorescent excitation, specifically within the wavelength ranges of 440–530 nm and 680–780 nm, served as the foundation for sucrose concentration detection. The nondestructive detection approach facilitated by FHIS minimized the sample damage while providing rapid and efficient analysis results, demonstrating significant potential for practical applications. This study employed advanced machine learning techniques, including VIP, SPA, and XGBoost to achieve feature extraction. Subsequent secondary feature analysis, combined with predictive models such as GBDT, RF, and LightGBM, enabled accurate sucrose prediction. Furthermore, the accuracy of various feature analysis methods and prediction models were systematically compared. These findings validate the efficacy of FHIS in predicting apple sucrose content and highlight its promising application in the nondestructive quality evaluation of agricultural products.

The study of Zhu et al. (Contribution 3) focused on the evaluation and prediction of fall-induced and collision-related damage in kiwifruit, establishing a multiscale finite element model for this purpose. Their work involved analyzing the geometric characteristics and mechanical performance parameters of kiwifruit using reverse engineering techniques and mechanical testing. By integrating these findings with the structural features of fruit tissues, they developed an interesting multi-scale finite element model that incorporated the skin, pulp, and core of fruits. Systematic simulations were performed to investigate the effects of varying drop heights, collision angles, and contact surface materials on kiwifruit damage. The accuracy of the model was validated, and the sensitivity prediction model for assessing the drop damage sensitivity of kiwifruit under different contact materials was also verified. This model serves as an effective tool for quantitatively analyzing drop damage in kiwifruit and provides a theoretical foundation for designing and optimizing loss-reduction strategies during centralized fruit harvesting.

The study conducted by Xin et al. (Contribution 4) integrated conventional physicochemical analysis with near-infrared spectroscopy (NIRS) nondestructive testing technology to propose an analytical model for determining the optimal harvest time of buckwheat. Initially, the study performed physicochemical analyses on the starch, protein, total flavonoid, and total phenol content in buckwheat grains across various growth cycles. Subsequently, spectral images corresponding to their harvest periods were captured using a near-infrared spectroscopy imaging system. Furthermore, the full-spectrum and characteristic-spectrum random forests (RF) for buckwheat were constructed by combining different preprocessing techniques and algorithms. Then, the optimal model for predicting the buckwheat harvest period was validated. Based on the results of the physicochemical analysis, it was found that the protein content in buckwheat grains with a 90-day growth cycle was relatively high. Among the preprocessing methods, the Standard Normalized Variate (SNV) method demonstrated the best performance, while the characteristic bands extracted using the IVSO algorithm were deemed more representative. The prediction accuracy of the established IVSO-RF model for the buckwheat harvest period reached 100%. This research not only ensures quality control for buckwheat but also holds significant potential for extension to the quality control of other crops, thereby demonstrating substantial value.

The study conducted by Wang et al. (Contribution 5) successfully fabricated a carbon black and carbon nanofiber composite (CB/f-CNF) via a straightforward one-step ultrasonic method. Owing to the high specific surface area of carbon black, this CB/f-CNF material can significantly enhance the electrical conductivity and porosity of carbon nanofibers, thereby facilitating electron transfer on the electrode surface and endowing the prepared composites with superior catalytic performance. Furthermore, the developed CB/f-CNF material was modified onto a glassy carbon electrode for signal amplification, leading to the construction of an amperometric sensor for the detection of environmental estrogen bisphenol A (BPA). It has been demonstrated that the electrochemical sensor based on CB/f-CNF exhibited remarkable linear response and high sensitivity towards bisphenol A, as well as achieving satisfactory recovery rates in the analysis of real samples such as meat and milk. This study provides a robust and promising analytical strategy for monitoring the migration of bisphenol A into food.
